# Social dominance explains within-ejaculate variation in sperm design in a passerine bird

**DOI:** 10.1186/s12862-017-0914-2

**Published:** 2017-03-04

**Authors:** Alfonso Rojas Mora, Magali Meniri, Sabrina Ciprietti, Fabrice Helfenstein

**Affiliations:** Laboratory of Evolutionary Ecophysiology, Institute of Biology, University of Neuchatel, Rue Emile-Argand 11, Neuchatel, Switzerland

**Keywords:** Sperm competition, Social hierarchies, Within-ejaculate variation, Soma vs. germline, Sperm morphology

## Abstract

**Background:**

Comparative studies suggest that sperm competition exerts stabilizing selection towards an optimal sperm design – e.g., the relative size and covariation of different sperm sections or a quantitative measure of sperm shape - that maximizes male fertility, which results in reduced levels of within-male variation in sperm morphology. Yet, these studies also reveal substantial amounts of unexplained within-ejaculate variance, and the factors presiding to the maintenance of such within-male variation in sperm design at the population level still remain to be identified. Sperm competition models predict that males should progressively invest more resources in their germline as their mating costs increase, i.e., the soma/germline allocation trade-off hypothesis. When access to fertile females is determined by social dominance, the soma/germline allocation trade-off hypothesis predicts that dominant males should invest less in the control of spermatogenesis. Hence, dominance should positively correlate with within-male variance in sperm design.

**Results:**

In support of this hypothesis, we found that dominant house sparrow males produce ejaculates with higher levels of within-ejaculate variation in sperm design compared to subordinate males. However, after experimentally manipulating male social status, this pattern was not maintained.

**Conclusions:**

Our results suggest that males might control variation in sperm design according to their social status to some extent. Yet, it seems that such within-ejaculate variation in sperm design cannot be rapidly adjusted to a new status. While variation in sperm design could result from various non-exclusive sources, we discuss how strategic allocation of resources to the somatic vs. the germline functions could be an important process shaping the relationship between within-male variation in sperm design and social status.

**Electronic supplementary material:**

The online version of this article (doi:10.1186/s12862-017-0914-2) contains supplementary material, which is available to authorized users.

## Background

Spermatozoa are one of the most morphologically diverse cells across animal taxa [1], with sizes ranging from 8 μm in the Hymenoptera *Meteorus sp.* [1] to 58 mm in *Drosophila bifurca* [2]. Such large variation in sperm morphology has presented a conundrum to sexual selection studies. Most of the efforts were put into identifying interspecific differences in the mean values of sperm morphology [3–9], though Ward [10] stressed that there is large intraspecific variation in sperm morphology. Further studies investigated such within-species, among-male variation in sperm traits [11–19], and many of them suggested that post-copulatory sexual selection is an important selective pressure in reducing the levels of among-male variation in sperm morphology. Although within-male variation in sperm has also been reported in some studies (e.g., [20]), what causes and maintains within-male variation in sperm morphology has been seldom explored.

There exist several non-mutually exclusive explanations as to why sperm can vary within ejaculates. A conflict between the diploid (i.e., the male phenotype) and the haploid genome (i.e., the spermatozoon phenotype) has been predicted to result in morphological variation [21, 22]. However, diploid genes are the main determinants of sperm morphology [23], and the optima for the diploid and haploid phenotype tend to be similar under strong sperm competition [21]. Production of large sperm numbers has been shown to be associated with more numerous errors in sperm production [24, 25], suggesting that inevitable developmental errors can partly maintain within male variation. It has also been suggested that sperm competition could lead to the evolution of different sperm phenotypes that play different roles in the ejaculate [19, 26], thus resulting in a mixed strategy ejaculate. Alternatively, Birkhead et al. [27] hypothesized that as the intensity of sperm competition is relaxed, males could afford lower sperm production control (e.g., higher morphological variation). Comparative studies have found support for the latter hypothesis [11, 13, 14, 28, 29], arguing that sperm competition exerts strong stabilizing selection towards optima in sperm design – e.g., the relationship between different sperm sections. While comparative studies stress the role of sperm competition in reducing intraspecific among-male variation, the levels of within-male variation do not seem to follow evident patterns (e.g., within-male variation does not match among-male variation [30]).

In species where the access to fertile females differs across males, theory predicts that non-favoured males should invest more resources in the production of high quality ejaculates (for reviews see [31, 32]). Further, sperm morphology and design have been correlated with male fertilizing success, and thus they might be important components of ejaculate quality (for reviews see [33, 34]). Two models predict that a continuous increase in costs to obtain a mate should select for continuously increasing resource investment in the production of high quality ejaculates, i.e., the soma/germline allocation trade-off hypothesis [35, 36]. In species where access to fertile females is determined by social dominance, we propose that the amount of within-male variation in sperm morphology will depend on male social status. Thus, based on the soma/germline allocation trade-off, we predict that as males are less dominant they should exhibit lower within-male variation in sperm morphology resulting from higher resource investment in sperm production control.

House sparrows *Passer domesticus* are socially monogamous birds with levels of extra-pair paternity ranging between 12-15% [37–40], and in this species, social hierarchies covary with male reproductive behaviours and male access to fertile females (e.g., mate-guarding, copulation attempts and success [41]). Moreover, a previous study on house sparrows reported a large amount of within-male variation, which, for some sperm traits, exceeded between-male variation [20]. Further, a more recent study found that dominant house sparrow males produce ejaculates of lower quality compared to those produced by males in the middle of the hierarchy, which results from lower resource investment in the protection of ejaculates against oxidative stress [42]. To test whether male dominance explains levels of within-male variation in sperm design, we maintained 60 wild male and 60 wild female house sparrows in outdoor aviaries, and investigated within-male variation in sperm design according to social rank after a 4-week acclimation period. To further test the causality of the observed patterns, we experimentally manipulated the social status of males in a way that optimized the number of males going up or down the hierarchy. In this study, we defined sperm design as the first principal component of a PCA using sperm head, midpiece, and flagellum length. Our measure of sperm design described spermatozoa in terms of the relative length of their midpiece and flagellum compared to the length of their head, the former two being negatively related to the latter.

## Results

### Before manipulating the social status

**Fig. 1:**
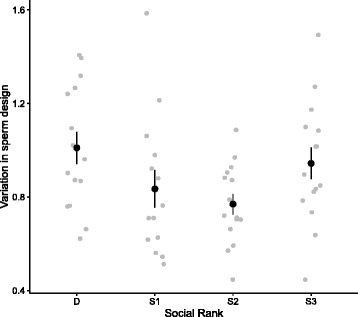
Relationship between variation in sperm design (scattered plot and mean ± SE) according to social rank *before* manipulating the social status

**Fig. 2:**
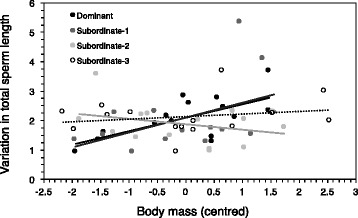
Relationship between within-ejaculate variation in total sperm length and body mass (centred) for males at different social ranks *before* manipulating the social status. The lines represent linear regressions. These relationships are maintained after removing a potential outlier (see text for details)

We found that males at different social ranks differed significantly in their within-ejaculate variation in sperm design (Fig. 1, Table 1a), with dominant and subordinate-3 males exhibiting larger within-ejaculate variance in sperm design. Social status did not explain variation in total sperm length (Table 1b). However, we found that variation in total sperm length varied non-additively according to the social status and the body mass (Fig. 2; rank × centred body mass, Table 1b), and this relation remained after removing a potential outlier due to a very light-weight subordinate-2 male with large variation in total sperm length (F_3,43.6_ = 3.26, *p* = 0.030). The relation between variation in total sperm length and body mass was significant and positive for dominant males only (slope ± SE; dominant males: β = 0.46 ± 0.11, t_10.1_ = 4.12, *P* = 0.002; subordinate-1 males: β = 0.47 ± 0.28, t_10.2_ = 1.71, *P* = 0.12; subordinate-2 males: β = -0.19 ± 0.24, t_12_ = 1.35, *P* = 0.20; subordinate-3 males: β = 0.06 ± 0.13, t_10.1_ = 0.43, *P* = 0.68), and this remained true when removing the potential outlier. Additionally, pairwise comparisons revealed that the relation between variation in total sperm length and body mass differed significantly between dominant vs. subordinate-2 males (F_1,22.1_ = 16.22, *P* = 0.006), dominant vs. subordinate-3 (F_1,22.1_ = 5.11, *P* = 0.034) and subordinate-1 vs. subordinate-2 males (F_1,20.1_ = 5.27, *P* = 0.033). However, after removing the potential outlier the difference between subordinate-1 vs. subordinate-2 was no longer statistically significant (F_1,19.1_ = 3.19, *P* = 0.10).

**Table 1:** LMMs investigating how social status affects within-ejaculate variation in sperm design or total sperm length

a) Sperm design				
Random effects	Estimates ± SE		Z	P
Aviary	0		0	1
Sampling date	0.011 ± 0.013		0.87	0.19
Fixed effects		F	df	P
Intercept	0.75 ± 0.08			
Social status^a^		3.38	3, 45	**0.027**
Dominant	0.053 ± 0.066			
Subordinate-1	-0.083 ± 0.067			
Subordinate-2	0.139 ± 0.066			
Body mass	0.021 ± 0.036	1.21	1, 45.6	0.28
Tarsus length	0.039 ± 0.077	2.29	1, 45.2	0.14
Social status x Body mass^a^		1.27	3, 45.1	0.30
Dominant	-0.020 ± 0.060			
Subordinate-1	0.075 ± 0.058			
Subordinate-2	-0.039 ± 0.057			
Social status x Tarsus length^a^		0.91	3, 45.3	0.44
Dominant	-0.088 ± 0.122			
Subordinate-1	0.131 ± 0.129			
Subordinate-2	0.038 ± 0.100			
b) Total sperm length				
Random effects	Estimates ± SE		Z	P
Aviary			1.03	0.15
Sampling date			0.63	0.26
Fixed effects		F	df	P
Intercept	2.12 ± 0.24			
Social status^a^		0.56	3, 34.4	0.64
Dominant	-0.024 ± 0.23			
Subordinate-1	-0.009 ± 0.24			
Subordinate-2	-0.26 ± 0.23			
Body mass	0.001 ± 0.14	3.45	1, 39.5	0.07
Tarsus length	0.16 ± 0.30	6.90	1, 45	**0.012**
Social status x Body mass^a^		4.34	3, 44.3	**0.009**
Dominant	0.45 ± 0.24			
Subordinate-1	0.51 ± 0.22			
Subordinate-2	-0.23 ± 0.22			
Social status x Tarsus length^a^		0.70	3, 43	0.56
Dominant	0.11 ± 0.48			
Subordinate-1	0.67 ± .50			
Subordinate-2	0.29 ± 0.39			

^a^Relative to subordinate-3 males. Values in bold indicate significance at α = 0.05; tests of random effects are based on Wald-Z; tarsus length and body mass were centred to allow for correct estimations of main “social status” effects

### After manipulating the social status

In order to address causal relationships between variation in sperm design and social status, we manipulated social ranks by shuffling males across aviaries. This resulted in many males sharing similar social ranks within the new groups, which prompted males to move up or down in the hierarchy. After manipulating the social status, we found that social status did not explain the level of variation in sperm design nor in total sperm length anymore (Table 2). We also found no correlation between body mass and variation in sperm design or total sperm length (Table 2).

**Table 2:** LMMs investigating how experimentally changing the social status affects the within-ejaculate variation in sperm design or in total sperm length

a) Sperm design				
Random effects	Estimates ± SE		Z	P
Aviary	0		0	1
Sampling date	0		0	1
Fixed effects		F	df	P
Intercept	-0.16 ± 1.19			
Initial status^a^		0.19	3, 39	0.90
Dominant	0.15 ± 0.16			
Subordinate-1	0.097 ± 0.178			
Subordinate-2	0.079 ± 0.144			
Final status^a^		0.70	3, 39	0.56
Dominant	0.181 ± 0.191			
Subordinate-1	0.011 ± 0.139			
Subordinate-2	-0.048 ± 0.156			
Body mass	-0.013 ± 0.026	0.10	1, 39	0.75
Tarsus length	0.056 ± 0.052	1.16	1, 39	0.29
Initial status x Final status^b^		0.33	9, 39	0.96
Dominant x dominant	-0.274 ± 0.243			
Dominant x subordinate-1	-0.150 ± 0.247			
Dominant x subordinate-2	-0.006 ± 0.252			
Subordinate-1 x dominant	-0.104 ± 0.290			
Subordinate-1 x subordinate-1	-0.109 ± 0.238			
Subordinate-1 x subordinate-2	-0.134 ± 0.241			
Subordinate-2 x dominant	-0.133 ± 0.247			
Subordinate-2 x subordinate-1	-0.075 ± 0.216			
Subordinate-2 x subordinate-2	0.018 ± 0.228			
b) Total sperm length				
Random effects	Estimates ± SE		Z	P
Aviary	0		0	1
Sampling date	0		0	1
Fixed effects		F	df	P
Intercept	3.548 ± 2.802			
Initial status^a^		0.16	3, 39	0.92
Dominant	-0.310 ± 0.375			
Subordinate-1	-0.517 ± 0.421			
Subordinate-2	-0.091 ± 0.339			
Final status^a^		0.63	3, 39	0.60
Dominant	-0.204 ± 0.452			
Subordinate-1	-0.253 ± 0.329			
Subordinate-2	-0.643 ± 0.369			
Body mass	-0.020 ± 0.062	0.10	1, 39	0.75
Tarsus length	-0.051 ± 0.122	0.17	1, 39	0.68
Initial status x Final status^b^		0.86	9, 39	0.57
Dominant x dominant	0.044 ± 0.574			
Dominant x subordinate-1	0.217 ± 0.583			
Dominant x subordinate-2	0.860 ± 0.594			
Subordinate-1 x dominant	0.271 ± 0.686			
Subordinate-1 x subordinate-1	0.304 ± 0.561			
Subordinate-1 x subordinate-2	1.054 ± 0.569			
Subordinate-2 x dominant	0.244 ± 0.583			
Subordinate-2 x subordinate-1	-0.246 ± 0.509			
Subordinate-2 x subordinate-2	0.132 ± 0.538			

^a^Relative to subordinate-3 males. ^b^Relative to subordinate-3 x subordinate-3 males. Tests of random effects are based on Wald-Z

How much individuals moved along the social ladder (difference in ranks after minus before) did not explain the difference in within-ejaculate variation between after and before the manipulation (Table 3), although there was a non-significant tendency for individuals moving up to produce less variable sperm in terms of total length (Table 3b). Males with longer tarsi produced less variable ejaculates in terms of total sperm length after the manipulation of the social status (β ± SE = -0.427 ± 0.15, Table 3b).

**Table 3:** LMMs investigating whether moving up or down the social ladder resulted in more or less within-ejaculate variation in sperm design or total sperm length

a) Difference (after – before) in the variation in sperm design				
Random effects	Estimates ± SE		Z	P
Aviary	0.010 ± 0.010		0.91	0.18
Sampling date	0.007 ± 0.012		0.58	0.28
Fixed effects		F	df	P
Intercept	1.115 ± 1.061			
Difference in social rank	-0.010 ± 0.025	0.18	1, 50.8	0.68
Body mass (after)	-0.027 ± 0.026	1.09	1, 47.3	0.30
Tarsus length	-0.026 ± 0.050	0.27	1, 46	0.60
b) Difference (after – before) in the variation in total sperm length				
Random effects	Estimates ± SE		Z	P
Aviary	0.107 ± 0.103		1.04	0.15
Sampling date	0.091 ± 0.141		0.64	0.26
Fixed effects		F	df	P
Intercept	10.258 ± 3.224			
Difference in social rank	-0.131 ± 0.075	3.04	1, 50.6	0.09
Body mass	-0.090 ± 0.080	1.26	1, 46.8	0.27
Tarsus length	-0.427 ± 0.150	8.08	1, 45.7	**0.007**

Values in bold indicate significance at α = 0.05; tests of random effects are based on Wald-Z

### Relation between within-male variance in sperm morphology and sperm performance

**Fig. 3:**
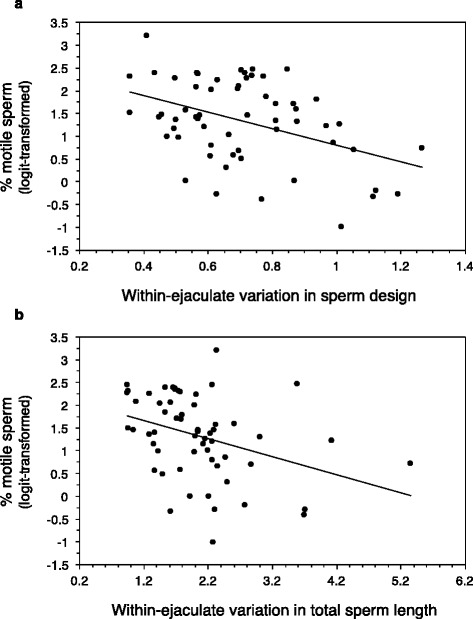
Relationship between the proportion of motile sperm in the ejaculate and (**a**) the within-ejaculate variation in sperm design or (**b**) the within-ejaculate variation in total sperm length *before* manipulating the social rank. The lines represent linear regressions

Before manipulating the social status, we found that the proportion of motile sperm correlated negatively with the amount of within-ejaculate variation in sperm design (β ± SE = -1.74 ± 0.59, F_1,49.6_ = 8.56, *P* = 0.005; Fig. 3a) and variation in total sperm length (β ± SE = -0.39 ± 0.14, F_1,57_ = 7.61, *P* = 0.008; Fig. 3b). However, sperm swimming ability was correlated neither with within-ejaculate variation in sperm design (β ± SE = -1.0.3 ± 0.68, F_1,55.7_ = 2.33, *P* = 0.13) nor within-ejaculate variation in total sperm length (β ± SE = -0.02 ± 0.17, F_1,56.8_ = 0.02, *P* = 0.90).

After manipulating the social status, we found that sperm swimming ability was negatively correlated with the amount of within-ejaculate variation in total sperm length (β ± SE = -0.63 ± 0.23, F_1,49.8_ = 7.34, *P* = 0.009). However, we found no correlation between percentage of motile sperm and variation in sperm design or total sperm length (β ± SE = -0.89 ± 0.62, F_1,56_ = 2.05, *P* = 0.16; β ± SE = -0.26 ± 0.26, F_1,56_ = 0.98, *P* = 0.33; respectively), nor between sperm swimming ability and within-ejaculate variation in sperm design (β ± SE = -0.17 ± 0.62, F_1,54.2_ = 0.08, *P* = 0.78).

## Discussion

In the present study, we found that the dominant and the most subordinate (subordinate-3) males exhibited greater within-ejaculate variation in sperm morphology and design than males at intermediate positions (subordinate-1 and subordinate-2) (Fig. 1). In addition, we found several negative correlations between sperm performance and within-ejaculate variation in sperm morphology and design (Fig. 3). Lastly, we found that heavier dominant and subordinate-1 males produced ejaculates with greater variation in sperm length compared to subordinate-2 and 3 males (Fig. 2). Previous studies have shown that males are able to adjust their ejaculate traits based on changes in their social environment (e.g., [43–45]), and previous studies on house sparrows have found that males can adjust their ejaculate quality in terms of percentage of motile sperm to changes in social status [42]. However, we did not observe any changes in the levels of variation in sperm design given the changes in social rank. While rapid changes in ejaculate function can be achieved by allocation of resources, which serve as protection for the ejaculate (e.g., antioxidants [42]), or which enhance ejaculate functioning (e.g., ATP [46]), adjustments in the spermatogenetic control might be more difficult to achieve after a single spermatogenetic cycle. The exact duration of a spermatogenesis cycle is unknown for our model species, but it is worth noting that it has been found to be between 11 and 15 days in non-passerine birds [47], and possibly less in passerine birds [48].

Birkhead and colleagues [27] found high levels of within-male variation in sperm morphology in male zebra finches, and they argued that the low levels of sperm competition in zebra finches may have led to relaxed pressures upon quality control processes in spermatogenesis, and thus the production of ejaculates with substantial variation in sperm morphology. While strong evidence for this hypothesis comes from interspecific comparative studies [11, 13, 14, 28], the production and maintenance of substantial intraspecific variation in sperm morphology is still not fully understood. Our results are consistent with the hypothesis that dominant males, which are likely to have a privileged access to females, are under relaxed sperm competition pressures, and consequently may loosen the quality control they exert on spermatogenesis, hence producing ejaculates with greater variance in sperm morphology and design.

We also found that, similarly to dominant individuals, fully subordinate males produced ejaculates with larger amounts of variance in sperm morphology and design. Although dominant and fully subordinate males share a common pattern of ejaculates variance in sperm morphology and design we propose that they may be subjected to different constraints or may respond to different selective pressures. Dominant males may produce ejaculates with larger variance in sperm morphology and design as a result of (i) larger sperm production demands to meet higher copulation rates, (ii) energy saving strategies, and/or (iii) strategic allocation of resources between somatic and reproductive tissues. On the other hand, fully subordinate males might be under energetic constraints (see below).

In Soay rams, it has been shown that dominant males have higher copulation rates throughout the reproductive season, and therefore run sperm depleted and lose out in sperm competition at the end of the season [49]. In house sparrows, dominant males have higher copulation rates [50, 51], and may thus face higher needs for sperm production. However, sperm production is costly [52], and increased sperm production may result in a larger number of errors [24, 25]. A larger number of production errors would be reflected in a lower proportion of motile sperm [25], and we indeed observed a negative correlation between within-male variation in both sperm design and total sperm length and proportion of motile sperm (Fig. 3). However, these correlations were not maintained after manipulating male social status (variation in total sperm length was correlated with sperm swimming ability instead). Thus, the hypothesis that increased variation in sperm design may result from a higher sperm demand in dominant, but not subordinate males still, deserves experimental investigations.

Alternatively, theory predicts that males should differentially invest in pre- vs. post-copulatory traits depending on the risk of sperm competition they face [31, 32, 35, 36]. The predictions of these models have been tested in various taxa, and evidence of such a pre- vs. post-copulatory investment trade-off exists [45, 49, 53, 54]. In house sparrows, a recent study showed that dominant males produce ejaculates of lower quality compared to ejaculates of subordinate males, while this reflects differences in the resources used to protect ejaculates from oxidative damage [42]. Birkhead and Immler [55] suggested that within-male variation in male design might result from individual differences of resource investment in spermatogenesis control, and thus the higher levels of within-male variation in sperm design by dominant males might be the result of differential investment in pre- vs. post-copulatory traits [32, 35, 36]. Strikingly, males at the lower end of the hierarchy also showed high levels of within-male variation in sperm morphology (Fig. 1). Males at the lower end of the hierarchy might face a high risk of sperm competition, and thus may be expected to reduce their ejaculate expenditure at any given copulation [36, 56]. Yet, males facing a high intensity of sperm competition are still expected to increasingly invest resources in post-copulatory traits [36], and thus they should be expected to invest in sperm production control. Alternatively, subordinate-3 males might be unable to predict the fertility status of females, and thus might benefit from producing ejaculates with various sperm types (e.g., mixed strategy ejaculates [20]). Finally, males at the lower end of the hierarchy may rather be under energetic constraints due to the physiological costs of subordination (e.g., higher allostatic loads [57]) and/or their lower ability to outcompete other males to access resources, and thus they might by unable to invest in spermatogenesis control.

In house sparrows, body mass affects male dominance [58], and smaller dominant males may face higher levels of male-male competition that may translate into higher risk of sperm competition. Further, smaller males seem to pay higher physiological costs when they are dominant [59]. Interestingly, we found that the lighter the males occupying higher social ranks (dominant and subordinate-1 males) were, the lower the variance in sperm morphology in the ejaculates they produced. This supports the idea that among dominant males the less competitive males may increase their investment in ejaculate quality to reduce variation in sperm morphology (Fig. 2), and hence increase the competitive ability of their ejaculates. However, whether slight differences in male competitiveness among higher ranking males result in differences in ejaculate quality remains to be experimentally tested.

## Conclusion

Different evolutionary processes may explain levels of morphological variation between taxa and within taxa [60], and sperm competition seems to cause stabilizing selection that leads to lower variation in sperm design across taxa (reviewed in the introduction). However, at the intraspecific level, we suggest that in species where males face different risks of sperm competition, they may flexibly adjust their investment in gamete production resulting in differences in the levels of within-male variation in sperm morphology that better fit their risks of sperm competition. Alternatively, males may invest equal amounts of resources in either producing small volumes of high quality ejaculates or large volumes of lower quality ejaculates. These two alternative hypotheses remain to be tested to better understand how high levels of intraspecific variation in sperm morphology and design are maintained. Furthermore, repeated samples of the same individuals would allow distinguishing within-male, between ejaculate variation from within-male, within-ejaculate variation to precisely address the various levels of variation. Lastly, experiments exploring the consequences of different levels of within-male variation in sperm design under sperm competition are needed to better understand male reproductive strategies.

## Methods

### Individuals and sampling

We trapped a total of 60 male and 60 female house sparrows using mist-nets in western Switzerland during the first two weeks of April 2014. From each bird, we measured body mass and tarsus length, and birds were assigned to 15 mixed outdoor aviaries at the Hasli Ethological Station (University of Bern, Switzerland) according to their body weight and an initial score of badge size. Thus, aviaries had on average birds of the same body weight and males with various badge sizes. After four weeks, all the females were transferred into a separate aviary, and we took a sperm sample from each male. We then collected a second sperm sample the day after, and a third sperm sample after 48 h from the last sample. This procedure ensured that any differences in sperm characteristics would be intrinsic differences in quality rather than differences due to depletion [49] or fresh sperm effects [61–63] (but see [64]), and only data collected after all males were manually depleted was used. Males were divided in three sampling bouts consisting of 5 aviaries, and each bout was processed 5 days apart.

To test the causality of the observed patterns, females were reintroduced to the aviaries and males were shuffled across aviaries according to their initial social rank. We maximized the number of positions that males could have gained or lost in the hierarchy (Additional file 1). Males were given three weeks to settle down the new hierarchical positions. The exact duration of spermatogenesis is unknown in house sparrows. However, spermatogenesis has been estimated to last between 11 and 15 days in non-passerine birds such as domestic fowls, Japanese quails and Barbary drakes [47], and a study by Bat & Maiti [48] on yellow-throated sparrows *Petronia xanthocollis* suggests that it may be shorter in passerine birds. We thus assumed that three weeks would cover at least one spermatogenesis cycle. At the end of these three weeks, we collected sperm samples following the same procedure as before.

### Social dominance

To determine males’ hierarchical positions, we filmed a total of 13 h before the manipulation and 10 h after the manipulation in each aviary. We observed the males interacting at the feeder, which consisted in a seed dispenser with two feeding stations, mounted on a plastic plate that was covered with a plastic mesh. Such feeder made any spilt seeds inaccessible, and thus birds had to compete for the two feeding sites at the seed dispenser. We removed the feeder 90 min before recording the videos, and then reintroduced the feeder together with a GoPro camera that was located at ca. 60 cm from the feeder. Using the dyads in each aviary (before the manipulation: 82 dyads per aviary on average, range 31-235; post-shuffling: 100 dyads per aviary on average, range 39-233), we estimated each male's David's score as a proxy for their social rank within each aviary [65].

### Sperm morphology and sperm performance

We gently massaged the males' cloaca to obtain ejaculates [66] that were collected in glass capillaries. 0.25 μL of ejaculate were diluted in 40 μL of preheated Dulbecco Modified Eagle Medium at 40 ° C and a video was recorded using a Toshiba CMOS HD camera (Toshiba co., Japan) mounted on a light microscope with 10× objective. We used an computer automatized sperm analyser plug-in [67] for ImageJ [68] to assess the percentage of motile sperm and mean values for VCL (curvilinear velocity, total distance travelled, μm/s), VAP (average path velocity, smoothed path using roaming average, μm/s), VSL (straight line velocity, distance from origin to end point, μm/s), linearity (LIN: VSL ⁄ VAP, path curvature), wobble (WOB: VAP⁄ VCL, side to side movement of the sperm head, also described as the oscillation of the actual trajectory about its average path), BCF (beat cross frequency, the frequency at which VCL crosses VAP, Hz), progression (PROG: average distance from origin on the average path during all frames analysed). Sperm having a VSL < 5 μm/s, a VCL < 15 μm/s, or a VAP < 10 μm/s were assumed to be either moved by drift or immotile. These estimates were based on 71 ± 37 sperm tracks (mean ± SD) per ejaculate.

Sperm motility (% of motile sperm) and sperm swimming velocity are determinant components of male fertility and sperm competitive ability [69, 70]. Therefore, we assessed sperm performance as (1) the percentage of motile sperm and (2) PC1 scores from a principal component analysis (with varimax rotation) of the other seven variables plus the number of sperm cells detected by the CASA software. This axis captured 61.4% of the variance and was positively correlated with VSL, VCL, VAP, WOB and PROG (0.73 < *r* <0.98, *P* < 0.0001), negatively correlated with BCF (*r* = -0.79, *P* < 0.0001) and not correlated with LIN and the number of tracks (-0.1 < *r* < 0.05, *P* > 0.30). Hence, this first principal component axis, hereafter referred to as “sperm swimming ability”, described sperm swimming fast and efficiently (fewer overall movements to achieve greater progression).

A small droplet from the ejaculate was immediately smeared with 10% formalin on a glass slide. From each slide, we took photos of ten intact sperm cells using the Nikon ACT-1 v2.70 software (Nikon Corporation, Japan) with a Nikon Digital Eclipse DXM1200 camera (Nikon Corporation, Japan) mounted on a Leica DM R microscope (Leica Microsystems GmbH, Germany) at 400× magnification and phase contrast 2. From sperm cells, we measured the straight head, midpiece, flagellum, and total length. Several studies have shown that ten sperm are enough to capture both the ejaculate mean and CV in sperm morphology [11, 71]. S.C. did all the measurements blind to male identity and social status. Additionally, each cell was independently measured twice to assess the measurement error (estimated at 4.89% for total length, 5.16% for head length, 5.03% for flagellum length, and 3.98% for midpiece length using variance component analyses), and the average of these two measurements was used for further analyses.

To summarize sperm morphological design, we performed a principal component analysis using sperm head, midpiece, and flagellum length, and extracted the first component. The percentage of variance explained by PC1 was 52.3%. This PC1 was positively correlated with midpiece length (*r* = 0.84, *P* < 0.0001) and flagellum length (*r* = 0.83, *P* <0.0001) and negatively correlated with head length (*r* = -0.40, *P* < 0.0001). Thus, a positive score along PC1, hereafter referred to as “sperm design”, described a spermatozoon with a long midpiece and a long flagellum, but a relatively short head (and vice-versa for negative scores). From each male, we then calculated the standard deviation for both sperm design (PC1 scores) and total sperm length. We chose to keep total sperm length apart, because this sperm trait has historically been, and still is, the main focus of research (e.g., [9, 30, 72, 73]).

### Statistical analyses

We used linear mixed models (LMMs) to test our hypotheses. In a first set of statistical models we modelled the standard deviation in sperm design or in total sperm length as a function of the social status, while including body mass and tarsus length (both centred on their social-status means) as covariates.

After we experimentally manipulated the social status of males, we ran a second set of similar models with standard deviation in sperm design or in total sperm length as the dependent variables, and both the initial and final social rank, as well as their interaction as explanatory variables. These latter models allowed us to account for potential effects of the initial social status on the plasticity in variation in sperm design and total length. The models also included tarsus length and body mass as covariates.

To maximally exploit the information contained in our dataset, we also ran a third set of models, encoding the amplitude of the difference between the initial and the final social rank as a continuous variable ranging from -3 to +3. It is to be noted that this encoding ignores the initial social status, and a similar amplitude, for instance -2, may be achieved by an initially dominant male becoming a subordinate-2 or by an initially subordinate-1 becoming a subordinate-3. However, the virtue of this coding is to account for the “social distance” moved up or down by each individual. In these models, we used the difference, after minus before the change in status, in the variation in sperm design or in total sperm length as the dependent variables. The models thus explore whether moving upwards or downwards the social ladder resulted in more or less within-ejaculate variation in sperm morphology. Models included body mass after manipulation and tarsus length as covariates.

Finally, to test the relationship between within-male variance in sperm morphology (design and total length) and sperm performance, we modelled the proportion of motile sperm and sperm swimming ability as a function of the variation in sperm design on the one hand, and the variation in total sperm length on the other hand both before and after social status was experimentally changed.

The proportion of motile sperm was logit-transformed to match normality. All models included the aviary and the sampling date as random factors, and models were estimated using a restricted maximum likelihood method for parameter estimation and a Kenward-Roger approximation of fixed effects degrees of freedom. Tests of fixed effects were based on SAS Type-II tests of hypothesis. We did not apply model selection to avoid inflating the type I error [74]. All the analyses were performed using ®SAS 9.4. The dataset and the scripts of the statistical analyses are provided as Additional files 2 and 3.

### Animal ethics

Animal manipulations were performed as quickly as possible to minimise stress. We recorded any injuries or anomalous behaviours that could indicate excessive pain or stress and would require euthanizing the animal according to our guideline. The veterinary office of the Canton Bern, Switzerland, after supervision and approval by the Cantonal ethical committee, authorized the experimental setup and detention conditions under licenses n° BE41/12 and WTH/g-525/14.

## Additional files

Additional file 1: David’s scores used as proxies for social rank. (XLSX 39 kb)
Additional file 2: Explicit variable names are to be found in comments. (XLSX 48 kb)
Additional file 3: Script of the statistical analyses run with SAS. (TXT 8 kb)
